# Different prognostic impact of *STK11* mutations in non-squamous non-small-cell lung cancer

**DOI:** 10.18632/oncotarget.6379

**Published:** 2015-11-25

**Authors:** Nicolas Pécuchet, Pierre Laurent-Puig, Audrey Mansuet-Lupo, Antoine Legras, Marco Alifano, Karine Pallier, Audrey Didelot, Laure Gibault, Claire Danel, Pierre-Alexandre Just, Marc Riquet, Françoise Le Pimpec-Barthes, Diane Damotte, Elisabeth Fabre, Hélène Blons

**Affiliations:** ^1^ INSERM UMR-S1147, Paris Sorbonne Cité Université, Paris, France; ^2^ Department of Medical Oncology, Hôpital Européen Georges Pompidou (HEGP), Assistance Publique, Hôpitaux de Paris, Paris, France; ^3^ Department of Biochemistry, Pharmacogenetic and Molecular Oncology Unit, Hôpital Européen Georges Pompidou (HEGP), Assistance Publique, Hôpitaux de Paris, Paris, France; ^4^ Department of Pathology, Hôpital Cochin, Assistance Publique, Hôpitaux de Paris, Paris, France; ^5^ INSERM U1138, Paris Sorbonne Cité Université, Paris, France; ^6^ Department of Thoracic Surgery, Hôpital Cochin, Assistance publique Hôpitaux de Paris, Paris, France; ^7^ Department of Pathology, Hôpital Européen Georges Pompidou (HEGP), Assistance Publique, Hôpitaux de Paris, Paris, France; ^8^ Department of Pathology, Hôpital Bichat, Assistance Publique, Hôpitaux de Paris, Paris, France; ^9^ Department of Thoracic Surgery, Hôpital Européen Georges Pompidou (HEGP), Assistance Publique, Hôpitaux de Paris, Paris, France

**Keywords:** lung cancer, STK11, LKB1, isoforms, prognosis

## Abstract

*STK11* is commonly mutated in lung cancer. In light of recent experimental data showing that specific *STK11* mutants could acquire oncogenic activities due to the synthesis of a short STK11 isoform, we investigated whether this new classification of *STK11* mutants could help refine its role as a prognostic marker. We conducted a retrospective high-throughput genotyping study in 567 resected non-squamous non-small-cell lung cancer (NSCLC) patients. *STK11* exons 1 or 2 mutations (STK11_ex1-2_) with potential oncogenic activity were analyzed separately from exons 3 to 9 (STK11_ex3-9_). *STK11*_ex1-2_ and *STK11*_ex3-9_ mutations occurred in 5% and 14% of NSCLC*. STK11* mutated patients were younger (*P = .01*) and smokers (*P< .0001*) *STK11* mutations were significantly associated with *KRAS* and inversely with *EGFR* mutations. After a median follow-up of 7.2 years (95%CI 6.8-.4), patients with *STK11*_ex1-2_ mutation had a median OS of 24 months (95%CI 15-57) as compared to 69 months (95%CI 56-93) for wild-type (log-rank, *P = .005*) and to 91 months (95%CI 57-unreached) for *STK11*_ex3-9_ mutations (*P = .003*). In multivariate analysis, *STK11*_ex1-2_ mutations remained associated with a poor prognosis (*P = .002*). Results were validated in two public datasets. Western blots showed that *STK11*_ex1-2_ mutatedtumors expressed short *STK11* isoforms. Finally using mRNAseq data from the TCGA cohort, we showed that a stroma-derived poor prognosis signature was enriched in *STK11*_ex1-2_ mutated tumors. All together our results show that *STK11*_ex1-2_ mutations delineate an aggressive subtype of lung cancer for which a targeted treatment through STK11 inhibition might offer new opportunities.

## INTRODUCTION

Molecular classification of non-small cell lung cancer (NSCLC) has a direct impact on treatment decisions. *STK11 (LKB1)* codes a serine/threonine kinase involved in the regulation of cell growth, polarity and motility [1]. Its inactivation has been initially described in human tumors associated with Peutz-Jeghers hereditary syndrome [2]. Subsequently, somatic *STK11* mutations have been reported in sporadic cancers [3] including NSCLC [4–6]. In Caucasian populations, mutation rate ranges from 25 to 30% in non-squamous NSCLC, whereas in Asians *STK11* is mainly inactivated through focal deletions [7, 8].

Although *STK11* is inactivated by a large spectrum of truncating mutation and behave as a tumor suppressor gene in different tumor models through mTOR inhibition [9–11], recent studies suggest that STK11 may also gain oncogenic properties. In lung cancer, a short STK11 isoform lacking the 124 N-terminal amino acids has recently been described as an oncogene [12]. This ΔN isoform is produced from internal translation using an alternative ATG initiation codon located in exon 3 in the context of a mutation occurring in exon 1 or 2 and lacks the nuclear localization signal and part of the kinase domain. In breast cancer, STK11 IHC showed the existence of different prognostic value between cytoplasmic STK11 and nuclear STK11. Indeed cytoplasmic STK11 was shown to interact with ERα/SRC/PI3K to stimulate the AKT pathway and was linked to a shorter survival [13]. These findings suggest that *STK11* may have tumor suppressor or oncogene functions. This dual mechanism may explain the absence of a clear association between *STK11* alterations and prognosis in lung cancer [14].

It prompted us to test the hypothesis that *STK11* exon 1-2 (*STK11*_ex1-2_) mutations resulting in a potential gain of oncogenic function through the expression of the ΔN-STK11 isoform have a different prognostic impact as compared to *STK11* exon 3-9 (*STK11*_ex3-9_) loss of function mutations.

## RESULTS

### Patients and genotyping characteristics

**Figure 1:**
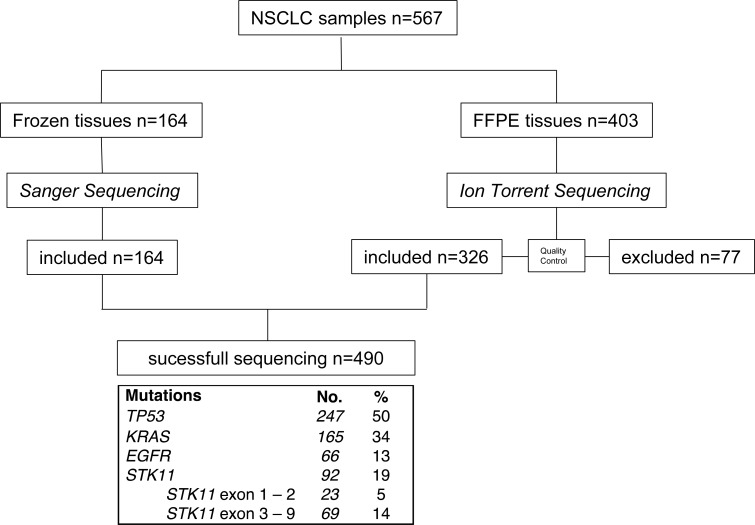
Flow chart of the study representing samples selection throughout the sequencing process Mutation frequencies are indicated. FFPE: formalin fixed paraffin embedded. NSCLC: non-small-cell-lung cancer, *STK11*_ex1-2_: *STK11* exon 1 and exon 2, *STK11*_ex3-9_: *STK11* exon 3 to exon 9.

Study flow chart and mutation frequencies are shown in Figure 1 and detailed in e-Table 1. Patient and tumor characteristics are detailed in Table 1. *STK11* mutated patients (*n = 92*) were younger (mean 58.6 ±11.3 vs. 61.9 ±11.2, *P = .01*) and more frequently smokers (96% vs. 80%, *P < .0001*). *STK11* mutations were associated with *KRAS* mutations (Odds Ratio = 2.0; *P = .01*) and inversely associated to *EGFR* mutations (Odds Ratio = 0.1, *P < .0001*). *STK11*_ex1-2_ and *STK11*_ex3-9_ mutations were found in 23 and 69 samples respectively. For these patients, clinical characteristics were similar (age, gender, type of surgery, smoking history, histological type, tumor stage (e-Table 2)). The association with *KRAS* remained true for *STK11*_ex1-2_ (Odds Ratio = 2.5, *P = .04*) as well as for *STK11*_ex3-9_ mutations (Odds Ratio = 1.9, *P = .02*).

**Table 1:** Summary of the patient and tumor characteristics and their prognostic effect (training series, n = 490)

Characteristic			Overall Survival					
			Univariate Cox model			Multivariate Cox model*		
	N	%	HR	95%CI	*P*			HR		95%CI		*P*
Gender								
Male	*327*	67	1					
Female	*163*	33	0.81	0.62-1.05	*.12*			
Age								
< 70 years	*366*	75	1				1	
≥ 70 years	*124*	25	1.48	1.13-1.93	*.005*	1.57	1.19-2.05	*.001*
Smoking history								
Present or former	*396*	83	1					
Never	*82*	12	0.89	0.62-1.23	*.48*			
NA	*12*	-						
Surgery								
Lobectomy	*427*	87	1				1	
Pneumonectomy	*36*	7	2.14	1.41-3.12	*.0006*	1.57	1.02-2.32	.04
Sub-lobar	*27*	6	1.41	0.81-2.26	*.21*			
Tumor stage								
I	*238*	49	1				1	
II	*118*	24	1.71	1.24-2.34	*.001*	1.59	1.15-2.19	*.006*
III	*111*	23	3.67	2.72-4.94	*< .0001*	3.59	2.64-4.87	*< .0001*
IV	*23*	5	3.62	2.05-6.00	*< .0001*	3.64	2.05-6.0	*< .0001*
Histological types								
Adenocarcinomas	*460*	94	1					
Other non-squamous NSCLC	*30*	6	0.86	0.50-1.37	*.54*			
Mutations								
Wild-type			1			1		
TP53	*242*		1.06	0.83-1.35	*.64*			
KRAS	*165*		1.38	1.07-1.77	*.01*	1.37	1.06-1.76	.02
EGFR	*66*		0.77	0.51-1.11	*.17*			
*STK11*_ex1-2_	*23*		2.00	1.17-3.17	*.01*	2.43	1.42-3.88	*.002*
STK11_ex3-9_	*69*		0.82	0.56-1.17	*.28*			

NA, not available; STK11_ex1-2_, *STK11* exon 1 and 2; STK11_ex3-9_, *STK11* exon 3 to exon 9; NSCLC, non-small-cell lung cancer; * STK11_ex3-9_ mutated patients were considered wild-type STK11 patients. The figure was marginally changed when STK11_ex3-9_ mutated patients are excluded from the analysis.

For 59 tumors, *STK11* mutations were associated with SNPs that allowed us to evaluate the loss of heterozygozity (LOH) at this locus. In 48 cases *STK11* mutations were associated with LOH (*N* = 45) or a second *STK11* mutation (*N* = 3), at similar frequency for *STK11*_ex1-2_ and *STK11*_ex3-9_ mutation types.

Distribution of mutations along the *STK11* locus revealed that disruptive mutations (nonsense, frameshift and splice mutations) tended to be over-represented in exons 1 and 2 (Odds ratio = 2.1; *P = .17*; e-Figure 1A and 1B). A significant over-representation of disruptive mutations in exons 1 and 2 was found in a dataset obtained from *STK11* next-generation sequencing across a wide variety of cancers (cBioportal [15], (Odds ratio = 3.34; *P = .004*; e-Figure 1C and D).

### STK11ex1-2 mutations are associated with a shorter overall survival

**Figure 2:**
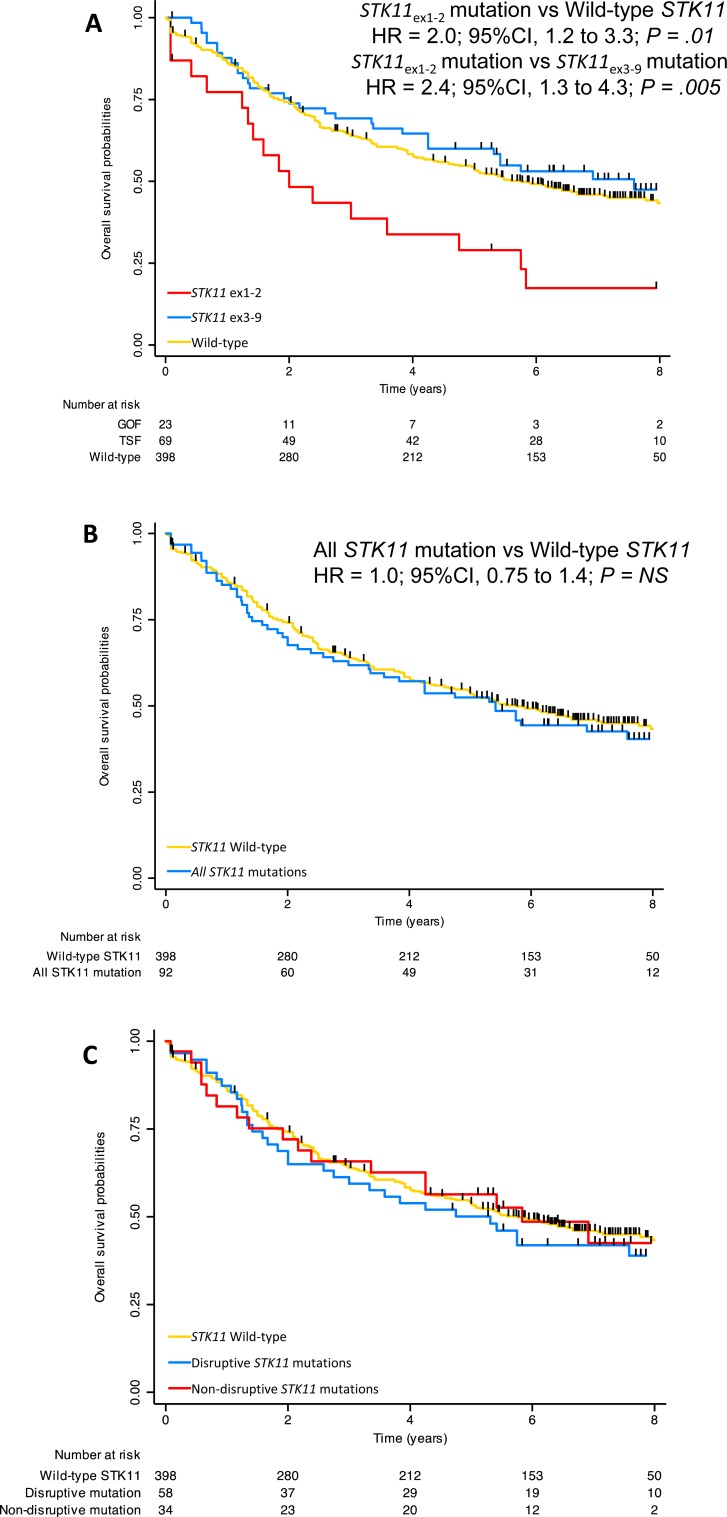
Overall survival according to ***STK11*** mutation status in present study. **A**. *STK11* mutations were categorized as *STK11*_ex1-2_ (located in exon 1 or 2), *STK11*_ex3-9_ (located in exon 3 to 9) or wild-type. **B**. *STK11* mutations considered all together as compared to wild-type. **C**. *STK11* mutations considered disruptive (nonsense or splice mutation, frameshift deletion) or non-disruptive (missense mutation or in-frame deletion).

**Figure 3:**
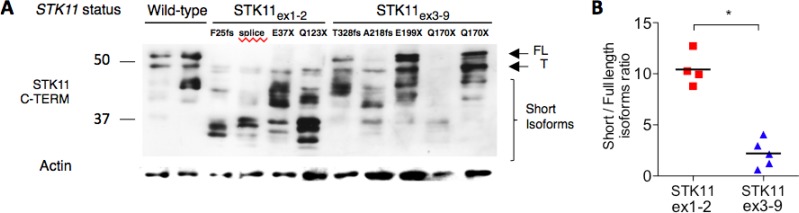
Assessment of STK11 isoforms according to ***STK11*** mutation status. **A**. Western-blot of tumor samples with *STK11* wild-type (WT) status (HP_6, HP_79), *STK11*_ex1-2_ (HD_88 p.F25fs, HD_179 c.291-2A>C, HD_340 p.Q37X, HD_428 p.Q123X) or *STK11*_ex3-9_ mutations (HD_294, HP_2, HD_61, HP_12, HP_237) revealed by a c-terminal anti-STK11 antibody Ley37D/G6 (1/1000). **B**. Full length (≥48kDa) and short isoforms ( < 48kDa) were quantified using ImageJ software and the short/long isoform ratios were calculated and compared among *STK11* mutation group using Mann-Whitney test. P-Values are indicated by *P < .05. Short STK11 isoforms are relatively overexpressed in lung tumor samples harboring *STK11*_ex1-2_ mutation. FL: Full length; T: Testis isoform, WT: wild-type.

After a median follow-up of 7.2 years (95%CI 6.8-.4) and 260 events, median OS was 5.6 years (95%CI, 4.8-6.9). Patients with *STK11*_ex1-2_ mutation had a median OS of 24 months (95%CI 15-57) as compared to 69 months (95%CI 56-93) in wild-type (log-rank, *P = .005*) and to 91 months (95%CI 57-unreached) in *STK11*_ex3-9_ mutations (log-rank, *P = .003,* Figure 3A). On the other hand, there was no difference in terms survival for patient with a *STK11*_ex3-9_ mutation as compared to wild-type *STK11* patients (log-rank, *P* = *.29*; Figure 2A). Five-year overall survival was 53% [49–58] in the entire population, 29% [14–51], 53% [49–59] and 60% [48–71] in *STK11*_ex1-2_ mutation, wild-type *STK11*, and *STK11*_ex3-9_ mutation, respectively. No difference in overall survival was noticed between *STK11* mutated samples and wild-type (HR = 1.03, 95%CI 0.75-1.40; Figure 2B). Similar results were found when classification was done according to the mutation type (disruptive vs. non-disruptive) (Figure 2C).

In multivariate analysis, *STK11*_ex1-2_ mutations were strongly associated with a poor prognosis (*P = .002*), as well as advanced tumor stage (*P < .001*), age >70 years (*P = .003*), pneumonectomy (*P = .03*) and *KRAS* mutations (*P = .01*) (Table 2).

**Table 2:** Validation of the pre-specified prognostic factors in two public series

Characteristic		Multivariate Cox model on PFS* Imielinski series (n = 128)				Multivariate Cox model on OS* TCGA series (n = 409)		
	N	HR	95%CI	*P*	N	HR	95%CI	*P*
Age								
< 70 years	*77*	1			*270*	1		
≥ 70 years	*54*	0.73	0.49-1.08	*.11*	*139*	1.53	1.05-2.22	.03
Tumor stage								
I	*75*	1			*218*	1		
II	*31*	1.06	0.66-1.67	*.79*	*96*	2.19	1.35-3.55	*.002*
III	*17*	2.15	1.14-3.80	*.02*	*74*	4.12	2.63-6.45	*< .0001*
IV	*8*	0.48	0.17-1.12	*.10*	*21*	3.01	1.40-5.88	*.006*
Mutations								
Wild-type		1				1		
*KRAS*	*36*	1.20	0.75-1.85	*.44*	*119*	1.22	0.79-1.86	*.36*
*STK11*_ex1-2_	*8*	2.65	1.11-5.60	*.03*	*22*	2.73	1.30-5.21	*.01*

We validated our results in two comparable independent public datasets (e-Table 3) [6, 15]. Available clinical endpoints were PFS in Imielinski series and OS in TCGA series. Median PFS in Imielinki series and OS in TCGA series were statistically shorter in patients with STK11_ex1-2_ mutated tumors (log-rank, *P* = *.003* and *P = .0098*, respectively*;* e-Figure 2A and 2B). In multivariate analysis, *STK11*_ex1-2_ mutations were associated with a poor prognosis in both series: HR = 2.65 (95%CI 1.11-5.61, *P = .03)* and HR = 2.73 (95%CI 1.30-5.21, *P = .01)* (Table 3). Moreover, PFS and OS for *STK11*_ex3-9_ mutated patients in Imielinski and TCGA series respectively were not statistically different as compared with *STK11* wild-type patients: HR = 1.16, (95%CI 0.58-2.10, *P = .64)* and HR = 1.28, (95%CI 0.72-2.11, *P = .38)*.

### Tumors with STK11_ex1-2_ mutations have increased short STK11 protein isoforms

We performed a western blot (WB) on available frozen tumor tissues from samples to semi-quantitatively measure the relative abundance of short/full-length isoforms. WB profiles differed between *STK11*_ex1-2_ and *STK11*_ex3-9_ mutated samples. Tumor harboring *STK11*_ex1-2_ mutations express short isoforms including the ΔN isoform (42kDa) as compared with wild-type and with *STK11*_ex3-9_ mutations (Figure 3A, 3B). Similar results were obtained with a second antibody (data not shown).

We aimed to address the relation between *STK11* mutation type and the expression of ΔN isoforms by a second approach. Therefore, we used mRNAseq data from the TCGA dataset to investigate whether *STK11* mutations could generate different types and levels of *STK11* transcripts. Although the total *STK11* expression was lower in tumor samples with *STK11*_ex1-2_ and *STK11*_ex3-9_ mutations (*P < .0001*) as compared to WT, the relative expression of exons 3 to 10 was higher in tumors with *STK11*_ex1-2_ mutations than in those with *STK11*_ex3-9_ mutations (*P = .003*, e-Figure 3).

### Gene expression profiles associated with STK11_ex1-2_ mutations

**Figure 4:**
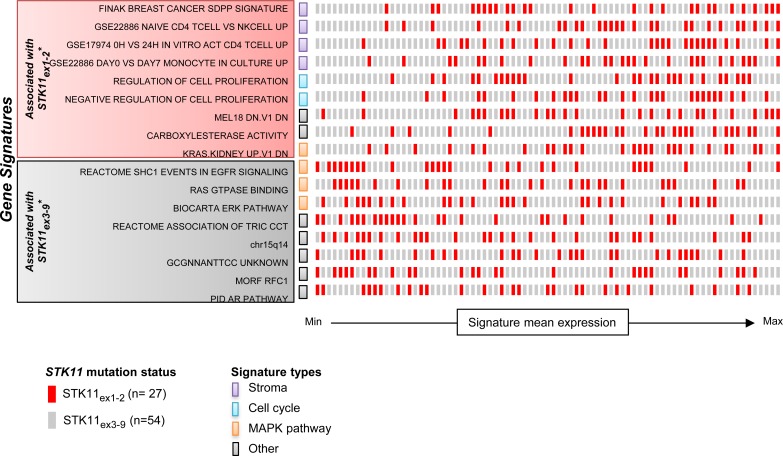
Gene signatures associated with ***STK11***ex1-2 and ***STK11***ex3-9 mutated tumor samples. Gene signatures were selected on the false discovery rate (FDR) of gene set enrichment analysis performed on mRNAseq from TCGA. Each row represent one gene set, and each square represent one sample colored according to its mutation type. The position of samples on the x-axis represents its ranked mean expression for the corresponding pathway. Gene signatures were categorized according to their main biological function: stroma, cell cycle, MAPK pathway or other. *All the gene sets had *FDR < .0001.* WT: wild-type; MAPK: mitogen-activated protein kinase.

mRNAseq data from the TCGA cohort was used to compare gene expression profiles on 20,501 genes between *STK11*_ex1-2_ and *STK11*_ex3-9_ mutated tumors. Gene expression patterns were statistically different in the two groups (under H1 proportion = .03). Gene set enrichment analysis in *STK11*_ex1-2_ mutated tumors identified as the most enriched signature a stroma-derived signature known to be associated with poor prognosis in breast cancer (Figure 4) [16]. *CXCL12* is the nodal gene associated with stroma signatures expressed in *STK11*_ex1-2_. Interestingly, mTOR related gene sets had similar expression levels in *STK11*_ex1-2_ and *STK11*_ex3-9_, confirming that both types of tumor share the loss of STK11 tumor suppressor activity and subsequent activation of the mTOR pathway. The RPPA analysis of the TCGA data found that AMPK phosphorylation, the direct target of *STK11*, was decreased in *STK11* mutated tumors as compared with wild-type (ANOVA, *P < .0001*), but was not different in *STK11*_ex1-2_ as compared with *STK11*_ex3-9_ (ANOVA, *P = .20*).

## DISCUSSION

Mutations occurring in tumor suppressor genes, such as *TP53*, have been shown to gain oncogenic functions [17]. Non-disruptive *TP53* mutations with potential gain of function have been associated with poor outcomes in multiple tumor types such as squamous cell carcinomas of the head and neck [18], chronic lymphocytic leukemia [19] and metastatic lung cancer [20].

Here, we describe similar findings and a potential dual impact of *STK11* mutations. The overall rate of *STK11* mutations (19%) was consistent with the one reported in Caucasians (15%-35%) [5, 7, 9, 14, 21]. We confirmed that *STK11* mutations were significantly associated with *KRAS* mutations [14]. The fact that *STK11*_ex1-2_ mutations were also associated with *KRAS* mutations might be related to a shared risk factor (tobacco) or an oncogenic cooperation between both alterations as for *BRAF* or *KRAS* with *PIK3CA* in lung and colorectal cancer [22, 23]. The identification of prognostic markers in lung cancer should not only consider the impact of one alteration but also the co-occurrence of other genetic events such as *KRAS* or *TP53* mutations. It was recently shown that *KRAS*/*STK11*; *KRAS*/*TP53*; *KRAS*/*TP53*/*STK11* are related to 3 different expression profiles with different pronostic impacts [24]. In our series, the prognostic value of *STK11* mutations was independent of the co-occurrence of a *KRAS* mutation.

We validated our pre-specified hypothesis that patients with *STK11*_ex1-2_ mutated tumors have a shorter survival as compared to others. The distribution of the *STK11* mutations along the gene coding sequence showed that disruptive mutations were more frequent in exon 1 and exon 2, as seen in a previous study [7]. This result is important as truncating mutations could favor the use of the alternative ATG initiation codon and the expression of the oncogenic ΔN isoform. Indeed, we showed in a subset of tumors for which proteins were available a significant increased expression of short STK11 isoforms using 2 different antibodies in tumors with *STK11*_ex1-2_ mutation as compared to tumors with *STK11*_ex3-9_ alterations_._ The relative overexpression of *STK11* exon 3 to 10 in *STK11*_ex1-2_ mutated tumors might indicate an active translation of these exons. We could not experimentally demonstrate that all mutations occurring in exon 1 or 2 led to a gain of STK11 function, nevertheless, patients with *STK11*_ex1-2_ mutated tumors share similar clinical characteristics and prognosis. In addition, the LOH frequency was similar in *STK11*_ex1-2_ and *STK11*_ex3-9_, which is consistent with previous works showing that full length STK11 inactivation is required for oncogenic functions of ΔN-isoforms.

The mechanisms and pathways affected by ΔN isoforms remains to be clarified. *In vitro* the ΔN isoform demonstrates oncogenic functions such as enhanced cell growth and loss of cell polarity. Here, gene expression of lung cancer with *STK11*_ex1-2_ mutations showed enrichment in stromal signatures. Strikingly, the top enriched signature was described to be associated with prognosis and recurrence in breast cancers [16]. In their original work, the authors demonstrated that silencing ΔN isoform using a ΔN-STK11 shRNA reduced tumor growth in a xenograft model with H460 lung cancer cell line that carries an exon1 mutation and expresses solely the ΔN isoform [12]. These data suggest that the inhibition of ΔN-STK11 in lung cancer with *STK11*_ex1-2_ mutations could be a potential therapeutic option. Up to now, therapeutic approaches for *STK11* mutant cancer were mainly conducted through the restoration of the mTOR pathway using mTOR inhibitors or phenformin, an analog of metformin, without taking into account the potential dual effect of *STK11* mutations [25].

Here, we demonstrate that *STK11*_ex1-2_ mutations with putative gain of oncogenic functions are associated with poor survival in resected non-squamous NSCLC and should be regarded as a potential therapeutic biomarker.

## MATERIALS AND METHODS

### Patients and tumors

The research was conducted according to the recommendations outlined in the Helsinki declaration and was approved by the Ethic Committee (CPP Ile-de-France II n°2008-136, 2008-133 and 2012 06-12). Consecutive patients with available tissue sample that underwent surgical resection in curative intent for primitive non-squamous NSCLC, at European Georges Pompidou Hospital (Paris, France) or Hôtel-Dieu (Paris, France), with curative intent from 2001 to 2006 were included (*N* = 567, number of event 306). Tumor pathological type and staging were determined according to World Health Organization criteria [26] on peut mettre l'OMS 2015 (cf papier mol immunitaire) and the revised lung cancer staging system [27]. Samples were collected and stored frozen or formalin fixed at time of surgery. An *a priori* power calculation was performed with the following hypotheses 1/ an estimated frequency of 6% of STK11 mutation occurring in exons 1 or 2 (cbioportal) in NSCLC 2/ an hazard ratio (HR) of 2 for the effect of these mutations on survival 3/ a failure of 30% in determining *STK11* status due to sequencing failure 4/ alpha two sided = .05 and power = .80. Therefore the estimated required number of events was 257.

### Genetic testing

Frozen samples (*N* = 164) were characterized by Sanger sequencing for *EGFR* (exons 18 to 21), *TP53* (exons 4 to 10), *KRAS* (exon 2) and the entire *STK11* coding region (exons 1 to 9) as previously described [28, 29]. FFPE samples (*N* = 403) were characterized using Next-Generation Sequencing (AmpliSeq Ion Torrent, Life Technologies) designed to amplify small DNA fragments, including *EGFR* (exons 18 to 21), *TP53* (exons 2 to 11), *KRAS* (exons 2 to 4) and *STK11* (exons 1 to 9). The panel used for NGS sequencing was a custom designed from Lifetechnologies (Ion AmpliSeq™ Custom DNA Panels). This design was composed of 83 amplicons and a coverage of 8772 bases, split into 2 primer pools and optimized for FFPE DNA detailed BED file is available at request. DNA input was 30 ng, libraries were prepared as recommended using the Ion AmpliSeq™Library Kit 2.0 on a Ion PGM™ System 2.0. The sequencing reads were processed using IonTorrent Suite V4.0. The Ion Torrent Variant Caller was used to call variants based on built-in “High-Stringency” settings. Called variants underwent subsequent quality control taking into account the coverage of each amplicon (≥100), the variant quality score (≥50) and the allele frequency (≥5%). Moreover, samples with high number of mutations (mainly transition due to FFPE) were considered as low quality when the number of variants by tumor was >50. Finally, 326/403 samples were considered adequate for mutation analysis with NGS (Figure 1). The rate of sequencing failure (*n* = 77/567, 14%) was inferior to our pre-specified hypothesis. Variant annotation was done with ANNOVAR. Variants listed in the NHLBI GO Exome Sequencing Project ESP (ESP6500SIV2 - July 2013) or the Exome Aggregation Consortium (ExAC) Version 0.3 (October 2014) with a frequency over 1/100,000 were excluded.

NGS results were validated by independent methods using Sanger sequencing (*STK11*), allele-specific real time PCR (*KRAS* and L858R *EGFR* mutation) and fragment analysis (*EGFR* deletions in exon 19 and insertions in exon 20) [30].

### Public dataset analysis

We analyzed two public dataset as validation series, including 128 patients clinically annotated for age and tumor stage with progression-free survival follow-up from the Imielinski series [6] and 409 patients annotated patients with overall survival follow-up from TCGA samples (Lung Adenocarcinomas, accessed on TCGA data portal 16^th^ April 2014) [4]. Mutation calls were downloaded from TCGA data portal and variations were annotated with ANNOVAR to exclude silent mutations and known polymorphisms. The transcriptome analysis were carried out using either an assortment of R system software packages including those of Bioconductor (V3.0) or original R codes. Gene expression and proteomic analyses were performed on mRNAseq and reverse phase protein array (RPPA) dataset downloaded at: http://gdac.broadinstitute.org/runs/stddata__2015_02_04/data/LUAD/20150204/.

RNA-seq raw counts were normalized with Trimmed Mean of M-values (TMM) [31], converted into log2 counts per million (logCPM), and analyzed using linear models and empirical Bayes methods implemented in limma package [32]. Resulting moderate T-tests were used to identify genes differentially expressed between groups of samples. The H1 proportion of T-tests over the set of measured transcripts was estimated using B Storey method : (1 - 2 × mean { if(pi > 0.5) then 1 else 0} probe set i:1..55K) [33]. To identify biological features associated with unsupervised samples partitions, 10 294 pathways collected from MSigDB (and related genes) were tested. GSA method was used to compare gene sets with sample groups [34]. For enriched pathways, tumor samples were sorted according to the mean rank expression of related genes. The expression of altered *STK11* RNA species that fit the profile of ΔN isoforms was evaluated using the exon mRNAseq dataset from TCGA. RNA-seq raw counts were normalized as previously described. The relative expression ratio of exon 3 to 10 / exon 1 and 2 was computed as the sum of reads mapping to exon 3 to exon 10 divided by the sum of reads mapping to exon 1 and exon 2.

### Western blot analysis

H358, H460 and H1993 cell lines were purchased at the American Type Culture Collection (ATCC). They were grown and stored at reception according to the specified instructions. For the present work, cell lines were genotyped and expected mutations were found for *KRAS* and *STK11*. These samples were only used to set up the *STK11* western blot conditions (data not shown). Frozen cancer cell lines and tumor tissues with different *STK11* status were extracted in RIPA buffer (Sigma-Aldrich). Two different C-Terminal anti-LKB1 antibodies were used: Ley37D/G6 (Santa Cruz) and D60C5 (Cell Signaling, data not shown). Different SKT11 isoforms were defined according to molecular weight (MW). Full length and testicular isoforms were defined by a MW >48kDa; short isoforms MW < 48kDa. Semi-quantitative evaluation of short / full isoforms was performed using ImageJ (NCBI). Isoforms ratios were compared among each STK11 tumor category using T-test.

### Statistical analysis

The clinical end point was overall survival (OS), calculated from the date of surgery to the date of death. Median follow-up was estimated with the use of the inverse Kaplan-Meier method. Proportions were compared using Fisher's exact test. Survival was estimated using Kaplan-Meier method and compared using log-rank test. Prognostic factors were first determined using univariate Cox proportional hazard model: gender, age, smoking history, tumor stage, histology, type of surgery, mutations: *TP53*, *KRAS*, *EGFR*, *STK11*_ex1-2_ and *STK11*_ex3-9_. The multivariate Cox proportional hazards model was undertaken on variables with univariate p-value < 0.10. All statistical tests were performed using JMP v10.0 (SAS), and a two-sided P-value less than 0.05 was considered statistically significant.

## SUPPLEMENTARY MATERIALS FIGURES AND TABLES




