# Supplementary material for: Different prognostic impact of STK11 mutations in non-squamous non-small-cell lung cancer
Source: Oncotarget. 2015 Nov 25;8(14):23831–40. doi: 10.18632/oncotarget.6379 (PMC5410347; doi:10.18632/oncotarget.6379)
Supplement: Supplementary file 1 [file oncotarget-08-23831-s001.pdf]

Different prognostic impact of *STK11* mutations in non-squamous non-small-cell lung cancer

Supplementary Material

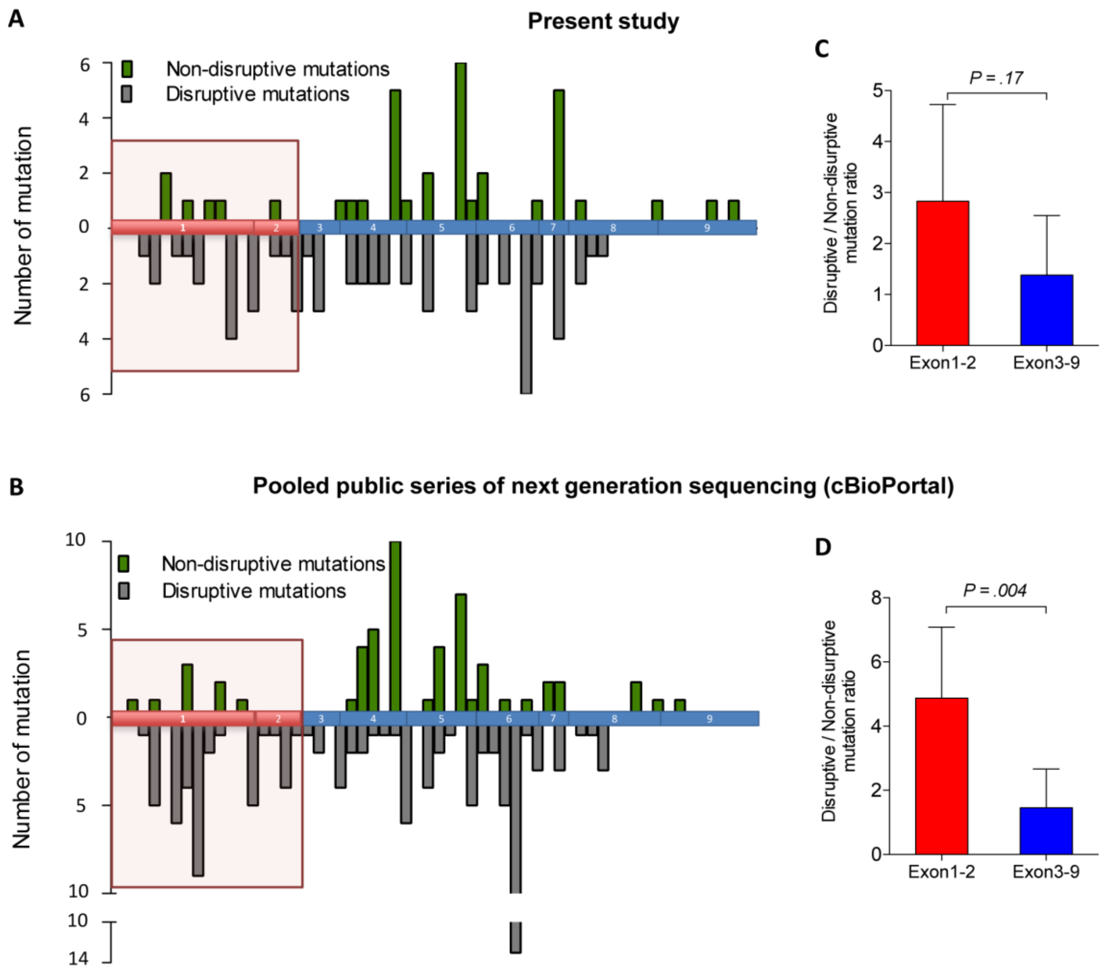

e-Figure 1. A and B. *STK11* mutations distribution along gene locus: Disruptive mutations (nonsense, frameshift indel, splice; grey box) vs. Non-Disruptive mutations (missense, inframe indel; green box). Each histogram reports the sum of *STK11* mutations within 22bp intervals. C and D. Disruptive mutation / non-disruptive mutation ratio in both series. Ratio is calculated for exons 1-2 and exons 3-9. Standard errors and chi^2 P-Values are indicated.

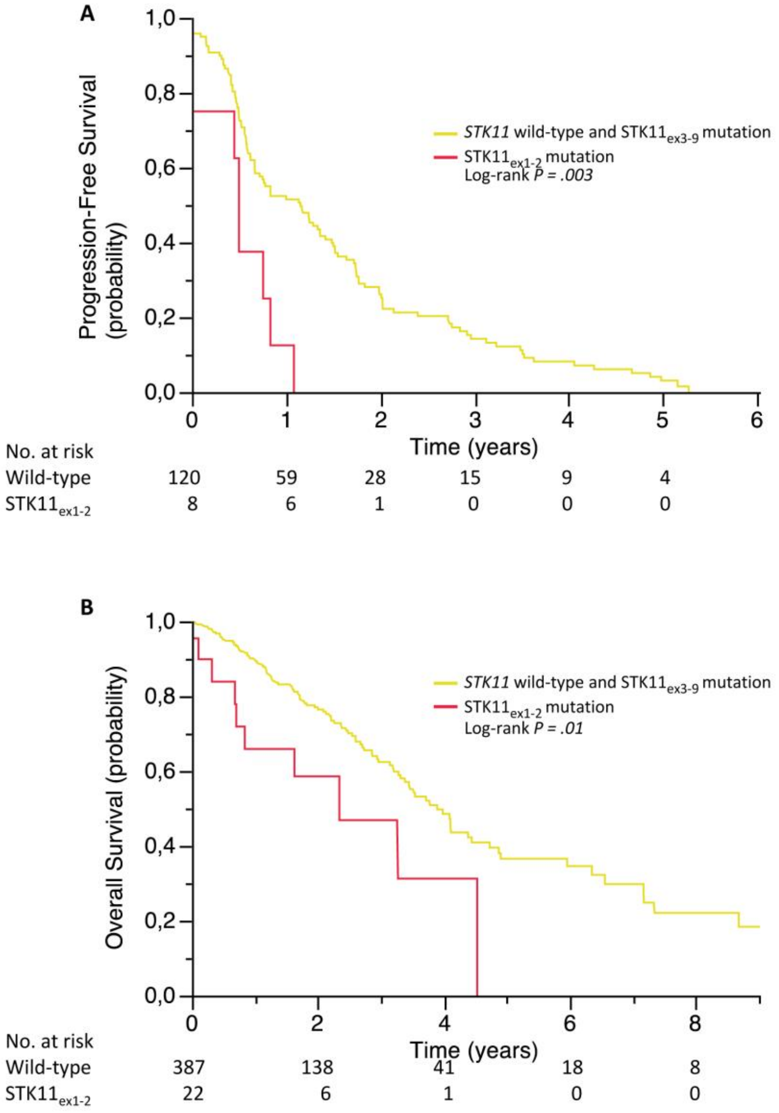

e-Figure 2. Progression-free survival and Overall survival according to STK11 mutation status in validation series: patients harboring *STK11*$_{ex1-2}$ mutations were compared to the others. A. Progression-free survival (N=128; Imielinski series). Patients with STK11$_{ex1-2}$ mutations had a median progression-free survival of 0.5 years (95%CI, 0 to 0.8) as compared to 1.2 years (95%CI, 0.7-1.4) for the others. B. Overall survival in the TCGA lung adenocarcinomas series (N=409). Patients with *STK11*$_{ex1-2}$ mutations had a median overall survival of 2.3 years (95%CI, 0.7-4.5) compared to 3.90 years (95%CI, 3.4-4.4) for wild-type patients.

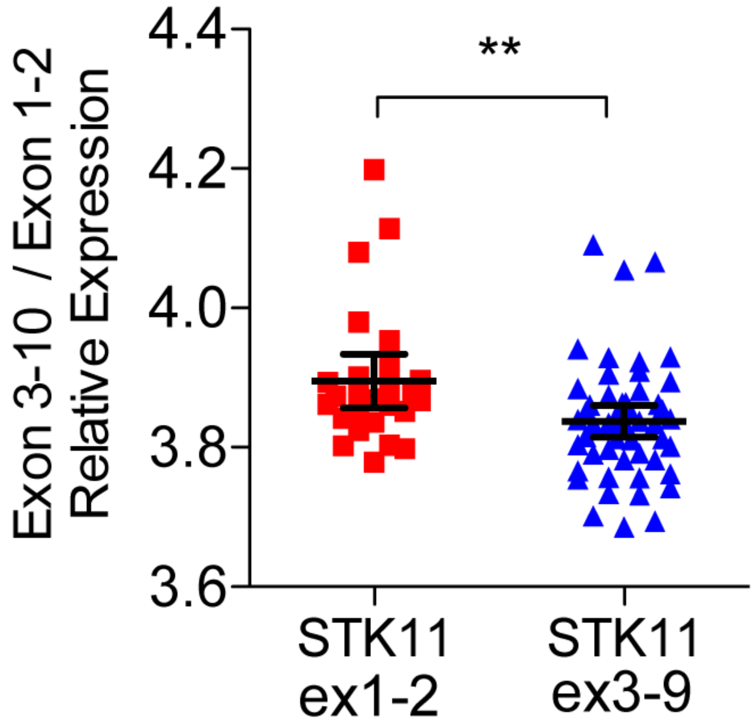

e-Figure 3. Expression of *STK11* exons 3 to 10 according to *STK11* mutation status. The exon expression dataset from TCGA mRNAseq was used. The relative expression ratio of exon 3 to 10 / exon 1 and 2 was computed as the sum of reads mapping to exon 3 to exon 10 divided by the sum of reads mapping to exon 1 and exon 2. The expression ratios were compared between *STK11* mutation groups using Mann-Whitney test. P-Values are indicated by $^{**}P < .005$.

e-Table 1. Annotated mutations found in our series.

e-Table 2. Patient and tumor characteristics in subgroups defined by *STK11* mutation status (training series, n = 490).

Characteristic	*STK11*$_{ex1\text{-}2}$ mutation (n = 23)		*STK11*$_{ex3\text{-}9}$ mutation (n = 69)		*STK11* wild-type (n = 398)		*P*
	N	%	N	%	N	%	
Gender							
Male	15	65	45	65	267	67	*.94*
Female	8	35	24	35	131	32	
Age							
Mean ± SD	60	±2.3	58	±1.3	62	±0.6	*.04*
Smoking history							
Present or former	21	91	67	97	308	20	
Never	2	9	2	3	78	77	*.002*
NA	0	0	0	0	12	3	
Surgery							
Lobectomy	20	87	62	90	345	87	
Pneumonectomy	2	9	6	9	28	7	*.57*
Non anatomic	1	5	1	2	25	6	
Tumor stage							
I	12	52	37	54	189	47	
II	6	26	13	19	99	25	*.88*
III	4	17	17	25	90	23	
IV	1	4	2	3	20	5	
Histological types							
Adenocarcinomas	23	100	67	97	370	93	*.18*
Other non-squamous	0	0	2	3	28	7	
Mutations							
TP53	13	57	34	49	200	50	*.83*
KRAS	12	52	31	45	122	31	*.01*
EGFR	0	0	1	2	65	16	*.0006*

NA, not available; GOF, gain of oncogenic function; TSF, tumor suppressive function; NSCLC, non-small-cell lung cancer. Proportions are compared using Chi-2. Means are compared using ANOVA.

e-Table 3. Patient characteristics of validation series (Imielinski and TCGA).

Characteristic	Imielinski series (N=128)		TCGA series (N=409)	
	No.	%	No.	%
Gender				
Male	*62*	48	*187*	46
Female	*66*	52	*222*	54
Age				
< 70 years	*77*	60	*270*	66
≥ 70 years	*51*	40	*139*	34
Tumor Stage				
I	*73*	57	*218*	53
II	*30*	23	*96*	24
III	*17*	13	*74*	18
IV	*8*	6	21	5
Histological type				
Adenorcarcinomas	*117*	91	*406*	100
Others	*11*	9	*0*	0
Mutations				
TP53	*60*	47	224	55
KRAS	*35*	27	119	29
EGFR	*19*	15	56	14
STK11	*21*	16	65	16
$STK11_{ex1\text{-}2}$	*8*	6	22	5
$STK11_{ex3\text{-}9}$	*13*	10	43	11

p<0.05 are in bold ; NA not available
